# Wernicke Encephalopathy in COVID-19 Patients: Report of Three Cases

**DOI:** 10.3389/fneur.2021.629273

**Published:** 2021-02-26

**Authors:** Marcus Vinicius Branco de Oliveira, Daniel Galera Bernabé, Sergio Irikura, Rodrigo Batista Irikura, Antônio Mendes Fontanelli, Marcus Vinícius Magno Gonçalves

**Affiliations:** ^1^Department of Neurology, Unimed Hospital of Araçatuba, Araçatuba, Brazil; ^2^Laboratory of Psychoneuroimmunology, Psychosomatic Research Center, Oral Oncology Center, Araçatuba Dental School, São Paulo State University (UNESP), Araçatuba, Brazil; ^3^Department of Medicine, University of the Region of Joinville (UNIVILLE), Joinville, Brazil

**Keywords:** Wernicke encephalopathy, COVID-19, encephalopathy, thiamine, acute respiratory distress, delirium

## Abstract

Encephalopathy has been frequently reported in patients with acute respiratory distress syndrome (ARDS) related to COVID-19, and its etiology remains undetermined. These patients display hypercatabolic state, weight loss, use of diuretics, and dialytic therapy, which represent risk factors for thiamine depletion. The diagnosis of Wernicke encephalopathy (WE) is challenging and based on risk factors for the depletion of thiamine. We reported three cases of patients with COVID-19-related WE treated with intravenous thiamine. All patients responded with immediate neurologic improvement, and two of them had accelerated ventilatory weaning. The cases reported suggest that thiamine deficiency could represent relevant etiology of COVID-19-related encephalopathy.

## Introduction

Encephalopathy is seen in more than 60% of COVID-19 patients with acute respiratory distress syndrome (ARDS) and is characterized by acute onset mental state alterations, varying from excessive drowsiness, delirium, torpor, and coma (1). Systemic inflammation or infection and other alterations (e.g., metabolic, electrolytic, and hypoxic) have been postulated as causes of encephalopathy in COVID-19 patients. In addition, the direct action of SARS-CoV-2 on the central nervous system (CNS) or even the role of indirect immune-mediated mechanisms has also been hypothesized (2). Although the occurrence of encephalopathies in patients with COVID-19 may be related to encephalitis, this mechanism seems to be responsible for only a small number of cases (2). Therefore, the etiology of most cases remains unknown.

Wernicke encephalopathy (WE) was described by Carl Wernicke in 1881, being characterized by the clinical triad of encephalopathy, ophthalmoparesis, and ataxia (3), secondary to the deficiency of thiamine. Recent studies have demonstrated a high prevalence (about 70%) of thiamine deficiency in critical patients with sepsis (4). In general, patients with severe COVID-19 have shown hyperinflammatory status (cytokine storm), high catabolic state, intense nutritional impairment with significant weight loss, frequent use of diuretics and dialysis therapy. Although these clinical characteristics are risk factors for reduced thiamine levels (4–6), the occurrence of WE in COVID-19 patients has not yet been shown. Here, we report for the first time the occurrence of WE in three critical COVID-19 patients, and their response to therapy with thiamine.

## Results

### Case 1

A 52-year-old man with a prior history of bariatric surgery, obesity, asthma, and tobacco use, was admitted to the hospital for treatment of COVID-19-related ARDS. The patient developed acute renal injury in hemodialysis and septic shock. A neurological evaluation was performed on the 33rd day of hospitalization and the 31st day of mechanical ventilation owing to difficulty of sedation removal and ventilation weaning associated with psychomotor agitation interposed with periods of consciousness level downgrades. On neurological examination, the patient displayed torpor and was not obeying commands. He had no motor response to painful stimulus in the four limbs, abolished oculocephalic reflex (ophthalmoplegia), diffuse hypoactive osteotendinous reflexes, without other abnormal findings. Laboratory exams revealed cerebrospinal fluid without alteration, including the absence of oligoclonal bands and normal IgG index. A brain CT scan was normal. An EEG did not show epileptiform paroxysms or electrographic seizures. In view of the presence of encephalopathy with ophthalmoplegia in a patient under risk of thiamine deficiency, the clinical diagnosis of WE was made, and intravenous thiamine was administered in a dose of 500 mg every 8 h for 5 d. Three d after starting treatment, the patient showed significant improvement of the neurologic condition with improvement in the level of consciousness, starting to obey commands, a complete improvement of ophthalmoplegia and the movement of the four limbs. After 3 months of follow-up, the patient was conscious, oriented, in spontaneous ventilation, with oral feeding, and walking independently.

### Case 2

A 51-year-old man with previous obstructive sleep apnea syndrome was hospitalized for COVID-19-related ARDS. The patient had septic shock, acute renal injury (necessitating hemodialysis), alveolar hemorrhage, and previously resolved atelectasis. He was examined on the 27th d in the Intensive Care Unit (ICU) (26th d of mechanical ventilation) for psychomotor agitation, aggressiveness, and mental confusion upon suspension of mechanical ventilation and sedation. Upon neurological examination, the patient alternated periods of psychomotor agitation and excessive drowsiness. He had isochoric pupils reactive to light, preserved ocular motor skills with horizontal nystagmus to looking to the right, slowing horizontal conjugate gaze in all directions, and brainstem reflexes maintained. The evaluation also revealed moderate cerebellar dysmetria in the upper extremities with bilateral intention tremor in the same segments, symmetrical movement of the four members, normative osteotendinous reflexes, and bilateral flexor cutaneous-plantar reflex. A brain CT showed no abnormalities, and similarly to the previous case, an EEG, demonstrated diffuse slowing suggestive of non-specific diffuse encephalopathy. Thiamine replacement (500 mg every 8 h) was performed, resulting in complete improvement in the level of consciousness and psychomotor agitation on the last day of treatment (5th d). There was also partial improvement of appendicular ataxia with the maintenance of nystagmus. In his last evaluation (56th d of follow-up), the patient was conscious on spontaneous ventilation, under oral feeding, oriented, without cerebellar disturbances, and walking with assistance.

### Case 3

**Figure 1:**
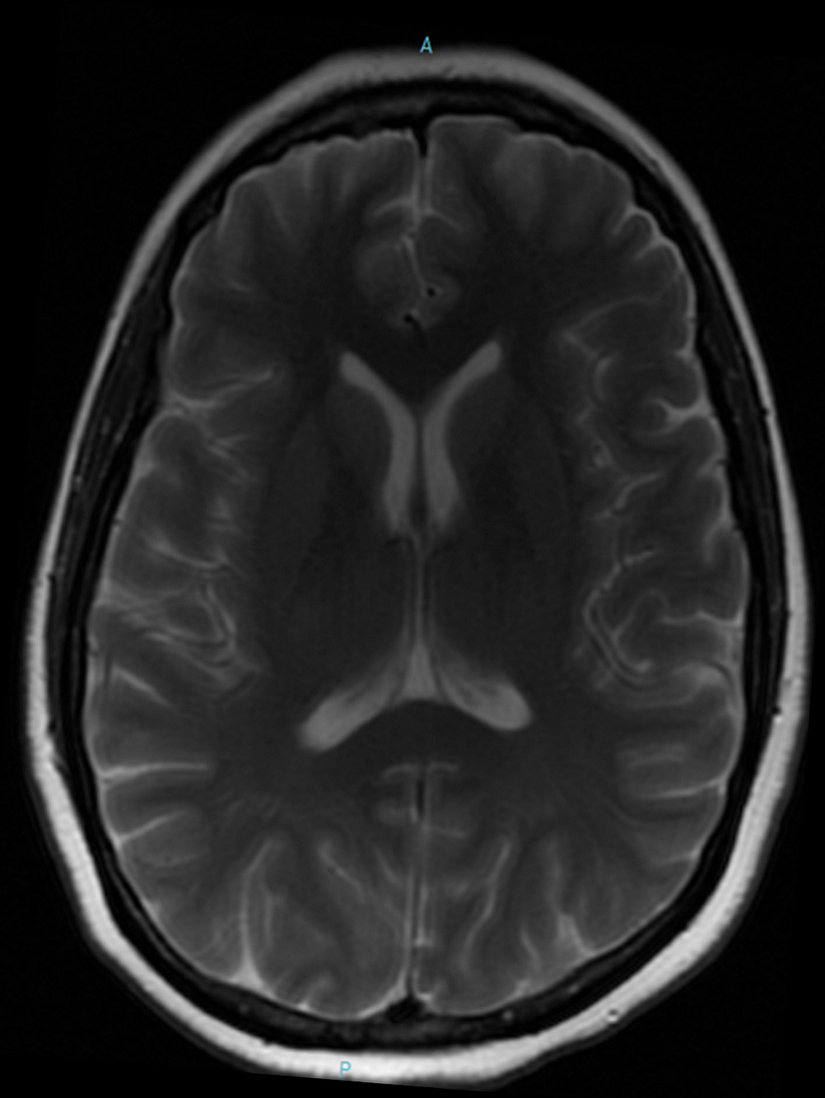
MRI T2 image of case 3 showing no brain abnormalities.

A 47-year-old woman was admitted to the hospital with a diagnosis of severe COVID-19-associated pneumonia. Her medical history was positive for hypothyroidism, hypoparathyroidism, and anxiety disorder. She was hospitalized in the ICU for 15 d and kept on mechanical ventilation for 5 d. The patient was subjected to neurological evaluation because of her report of mental confusion, visual and auditory hallucinations, insomnia, and psychomotor agitation soon after sedation removal. After discharge to the infirmary, she showed spontaneous improvement of agitation, though with persistent complaint of short-term memory problems and executive dysfunction. The patient also complained of a burning pain in inferior members and imbalance when walking, being able to walk only with assistance. Upon neurological exam, the patient was alert, conscious, and temporally disoriented. The oculomotor exam revealed paresis of the conjugated downward gaze without nystagmus. She also had discrete cerebellar dysmetria of the upper extremities, ataxic gait needing assistance to walk, compromised static balance, and a positive Romberg test. MRI (Figure 1) and EEG exams showed no abnormalities. Cerebrospinal fluid analysis was also within the normal range, including the absence of oligoclonal bands and normal IgG index. The patient underwent intravenous thiamine therapy (500 mg, 8/8 h) for five d. After two d of treatment, there was partial improvement of memory, executive dysfunction, ophthalmoparesis, cerebellar dysmetria in the upper extremities, and gait ataxia. Thiamine treatment also promoted complete improvement of static balance, insomnia, and neuropathic pain in the lower extremities, though maintaining positive Romberg test. After 20 days from discharge, the patient was reassessed on an outpatient basis and showed discrete memory and executive function complaints, without impairment of the performance of daily life activity and maintenance of functional independence. The patient displayed complete improvement of gait ataxia, a negative Romberg test, oculomotor skills with discrete paresis of the right trochlear nerve, and reported discrete allodynia in the lower extremities.

## Discussion

We report three patients with COVID-19 related ARDS who had WE syndrome characterized by mental state alteration, ophthalmoparesis, and ataxia, in addition to peripheral neuropathy symptoms. All patients were treated with intravenous thiamine (500 mg dose every 8 h for 5 days) with significant improvement of neurological outcome. Before diagnosis, they spent a long time in the ICU under mechanical ventilation. All patients presented septic shock and needed diuretics. Two of them had acute renal injury, requiring dialytic therapy. Although collected from few clinical cases, the data reinforce the association of WE with several risk factors for thiamine depletion shared by critical COVID-19 patients. Expressive clinical improvement was observed in all patients after the 3rd d of the infusion of thiamine. Replacement of thiamine induced an improvement of the level and content of consciousness and also of ophthalmoparesis, gait ataxia, cerebellar dysmetria, and neuropathic pain. All patients had hospital discharge showing excellent neurologic outcome, orientation, ability to walk (two of them without assistance), and independence for basic daily life activities (Table 1).

**Table 1:** Neurological manifestations, risk factors for thiamine deficiency, and outcomes in the patients with COVID-19-associated Wernicke encephalopathy.

**Case**	**1**	**2**	**3**
Neurological manifestations	Torpor, ophthalmoparesis, tetraparesis, hyporeflexia.	Delirium, dysmetria and action tremors.	Delirium, memory disturbance, hallucinations, insomnia, ophthalmoparesis, gait ataxia, neuropathic pain
Septic shock	Yes	Yes	Yes
Use of diuretics	Yes	Yes	Yes
Acute renal injury/dialytic therapy	Yes/hemodialysis	Yes/hemodialysis	No
Length of ICU hospitalization^*^	33 days	27 days	15 days
Length of mechanical ventilation^*^	31 days	26 days	5 days
Neurological outcome after intravenous thiamine	Improvement in level of consciousness and ophthalmoparesis	Improvement in level of consciousness and ophthalmoparesis	Complete improvement of insomnia, neuropathic pain, and ataxia and partial improvement of memory.

* *Until diagnosis of Wernicke encephalopathy; ICU, Intensive Care Unit*.

There are another 15 patients with COVID-19-associated encephalopathy being treated at our service. Although most of them lacked the classic findings of WE, they also were treated with thiamine infusion with improvement of neurological outcomes, and the treatment has allowed successful early ventilator weaning in these patients. Viral or autoimmune encephalitis are less probable diagnoses for the patients reported since findings of cerebrospinal fluid inflammation were absent. Vascular etiologies and non-convulsive *status epilepticus* were ruled out based on the non-specific findings in neuroimaging exams and an electroencephalogram, respectively. Moreover, residual sedative effect is unlikely, because neurological evaluation was performed more than 48 h after sedation removal.

Thiamine plays a key role in cellular oxidative metabolism and acts as a cofactor of enzymes (e.g., pyruvate desidrogenase) responsible for energy homeostasis (4). Given the reported cases we hypothesized that high catabolic states associated with COVID-19-induced cytokine storm could accelerate thiamine depletion. In addition, the depleter effect of furosemide and hemodialysis would trigger deficiency of thiamine, reducing the conversion of pyruvate to acetyl-coenzyme and promoting a decline in ATP production, as well as the neurological manifestations (4). The diagnosis of WE is clinical, based on both the triad of encephalopathy, ophthalmoparesis, and ataxia and on the risk factors for the depletion of thiamine (6). The sensitivity and specificity of MRI in Wernicke Encephalopathy are 50 and 93%, respectively (7). Normal findings do not exclude the diagnosis (7). The absence of a precise serum dosage of thiamine, as well as the occurrence of the clinical triad classic in only 10% of the patients, makes the WE diagnosis challenging (6). It is estimated that only 15% of the cases are diagnosed before death (6). Early treatment of WE leads to rapid and significant recuperation of the patients, reducing the risk and severity of possible neurocognitive sequelae, in addition to functional improvement (6).

In view of the clinical findings and the excellent therapeutic response in the cases reported, it becomes imperative that attention be given to the diagnosis of WE in critical patients with SARS-CoV-2 infection. In addition, due to its effectiveness and low cost, thiamine infusion should be considered for the treatment of COVID-19-associated encephalopathies.

## Data Availability Statement

The original contributions presented in the study are included in the article/supplementary material, further inquiries can be directed to the corresponding author/s.

## Ethics Statement

Written informed consent was obtained from the patients for publication of the manuscript.

## Author Contributions

MB: research project organization, conception of project, manuscript preparation (writing of the first draft, review), including medical writing for content, and clinical evaluation of patients and recruitment of patients. DB and MG: manuscript preparation, review, and critique, including medical writing for content. SI, RI, and AF: manuscript preparation including review of manuscript. All authors contributed to the article and approved the submitted version.

## Conflict of Interest

The authors declare that the research was conducted in the absence of any commercial or financial relationships that could be construed as a potential conflict of interest.
